# CD1a and skin T cells: a pathway for therapeutic intervention

**DOI:** 10.1093/ced/llad460

**Published:** 2024-01-04

**Authors:** John H Ye, Yi-Ling Chen, Graham Ogg

**Affiliations:** MRC Translational Immune Discovery Unit, MRC Weatherall Institute of Molecular Medicine, University of Oxford, Oxford, UK; MRC Translational Immune Discovery Unit, MRC Weatherall Institute of Molecular Medicine, University of Oxford, Oxford, UK; Chinese Academy of Medical Sciences Oxford Institute, University of Oxford, Oxford, UK; MRC Translational Immune Discovery Unit, MRC Weatherall Institute of Molecular Medicine, University of Oxford, Oxford, UK; Chinese Academy of Medical Sciences Oxford Institute, University of Oxford, Oxford, UK

## Abstract

The CD1 and MR1 protein families present lipid antigens and small molecules to T cells, complementing well-studied major histocompatibility complex–peptide mechanisms. The CD1a subtype is highly and continuously expressed within the skin, most notably on Langerhans cells, and has been demonstrated to present self and foreign lipids to T cells, highlighting its cutaneous sentinel role. Alteration of CD1a-dependent T-cell responses has recently been discovered to contribute to the pathogenesis of several inflammatory skin diseases. In this review, we overview the structure and role of CD1a and outline the current evidence implicating CD1a in the development of psoriasis, atopic dermatitis and allergic contact dermatitis.

## Teaser

The CD1 and MR1 protein families present lipid antigens and small molecules to T cells, complementing well-studied major histocompatibility complex–peptide mechanisms. The CD1a subtype is highly and continuously expressed within the skin, most notably on Langerhans cells, and has been demonstrated to present self and foreign lipids to T cells, highlighting its cutaneous sentinel role. Alteration of CD1a-dependent T-cell responses has recently been discovered to contribute to the pathogenesis of several inflammatory skin diseases. We overview the structure and role of CD1a and outline the current evidence implicating CD1a in the development of psoriasis, atopic dermatitis and allergic contact dermatitis.

## Introduction

Inflammatory skin diseases are common and are associated with other systemic diseases that have a marked impact on quality of life. T-cell infiltrates are well known to be associated with such diseases and we now know that inhibition of T-cell-derived cytokines and their receptors can offer dramatic therapeutic benefit. However, we still know surprisingly little about what the T cells are responding to. This is an important question in terms of defining disease pathogenesis and new approaches to treatment. Traditionally, the activation of T cells has been thought to be dependent on recognition of cognate major histocompatibility complex (MHC)–peptide complexes by the T-cell receptor (TCR). However, in recent decades, MHC-independent T-cell activation pathways have also been identified involving nonpolymorphic molecules such as CD1 and MR1, which present lipid antigens and small molecules.^1^ These antigen-presenting molecules are relatively nonpolymorphic, opening up the possibility of discovering general rules that can be applied to all patients.^2^

## CD1 molecules

The CD1 class of proteins consists of five isoforms (CD1a–e) that are subclassified into three different groups based on structure, function and cellular localization.^3^ Specifically, group 1 (CD1a, CD1b and CD1c), and group 2 (CD1d) CD1 molecules are involved in the presentation of lipid antigens. However, the expression of group 1 molecules is largely limited to antigen-presenting cells (APCs) and thymocytes, whereas CD1d is additionally expressed by other haematopoietic and epithelial cells.^1^ In contrast to the aforementioned CD1 molecules, CD1e is involved in lipid processing and loading rather than antigen presentation.^4^ CD1 molecules have been implicated in a broad range of disorders such as cancer, infections, autoimmune disorders and allergies.^5–9^

Within the skin, CD1a is highly expressed by cutaneous mononuclear phagocytes such as Langerhans cells and subsets of dermal dendritic cells.^10–12^ In addition to CD1a, Langerhans cells also express langerin (CD207), a pattern recognition receptor, which was found to assist CD1a antigen loading.^13^ Interestingly, CD1a expression can also be induced in response to cytokines and other mediators on innate lymphoid cells type 2 (ILC2s), an innate homologue to adaptive T-helper 2 cells,^14^ and a population of activated BDCA-2^+^ skin-infiltrating dendritic cells.^12^ Therefore, CD1a is well placed to act as a sentinel to detect breaches in barrier integrity and may be a key driver of associated pathology. Beyond the skin, CD1a expression has been identified in subsets of cells in the nasal mucosa, lungs, gut, conjunctiva, cervix and other tissues.^15–24^

## CD1a-relevant lipids

CD1a regulates T-cell activation via the presentation of a diverse range of endogenous and exogenous CD1a lipids.^10,25,26^ The lipid-dense epidermis and sebum provide a continuous pool of CD1a antigens, including wax esters, squalene, fatty acids, sphingomyelins, sulfatides and triacylglycerides.^27,28^ The capture of lipids by CD1a is thought to occur at multiple locations: at the cell surface, during translocation through the endoplasmic reticulum and Golgi secretory pathway, and following internalization into the early endosomal pathway.^29,30^ This allows CD1a to sample a broad range of ligands and drive immune responses. The selectivity of lipids captured by CD1a is partly mediated by its unique antigen-binding cleft, consisting of two pockets (A′ and F′). The F′ pocket is connected to the extracellular environment via the F′ tunnel permitting antigen loading, whereas the roofed A′ pocket acts to regulate antigen size that mostly accommodates lipids with acyl chains containing 32–42 carbons.^31,32^ Recent advances in identification and quantitation of lipids eluted from CD1 isoforms have shown remarkable patterns of shared and unique molecular species that will help form the basis of future analyses and disease associations.^33^

## CD1a-mediated T-cell activation

**Figure 1:**
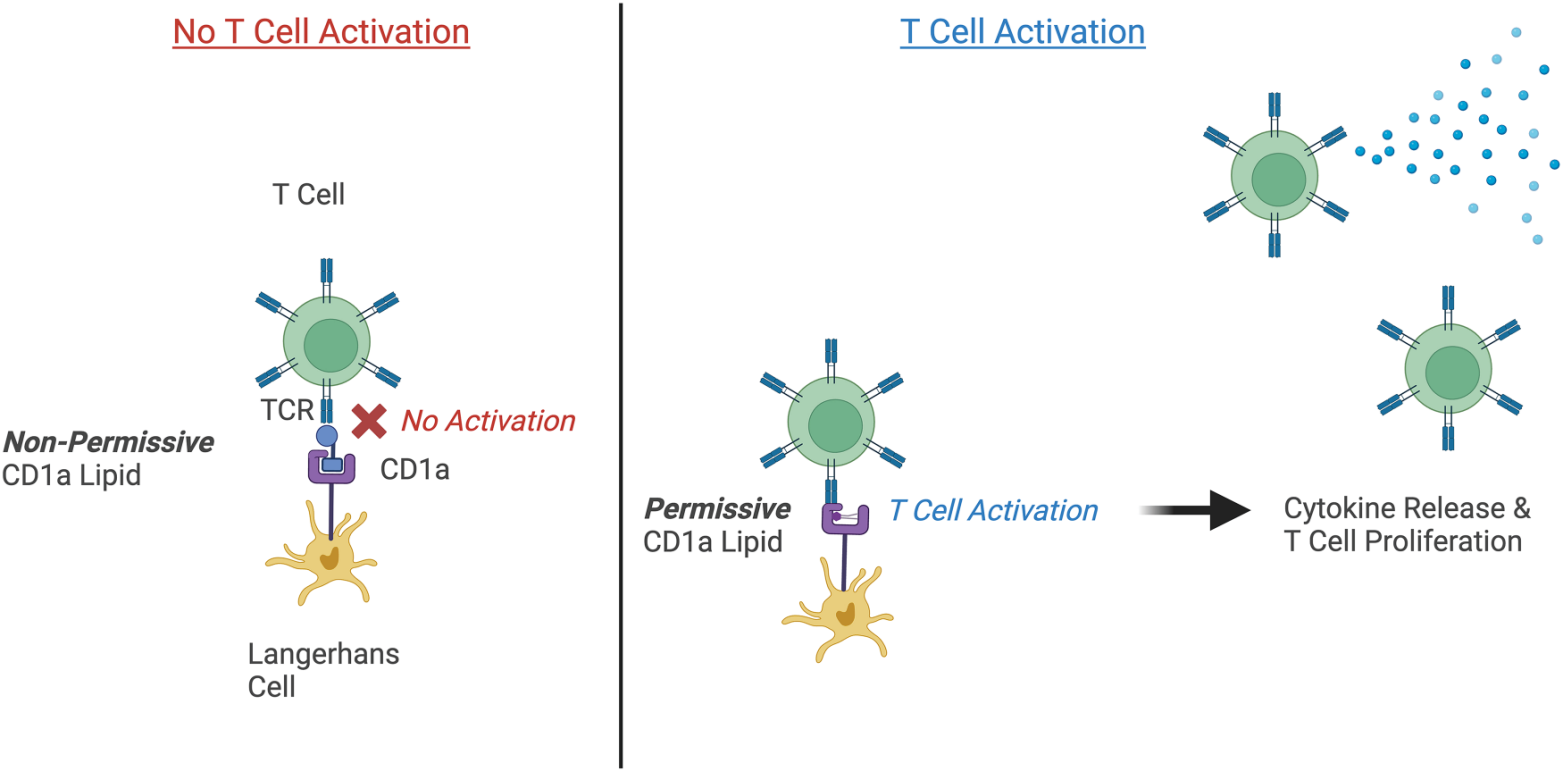
Absence of interference model. For some forms of CD1a-autoreactive T-cell activation to occur, direct engagement of the T-cell receptor (TCR) with the outer surface of CD1a is required. Therefore, some nonpermissive CD1a lipids that protrude beyond the CD1a binding cleft, physically inhibit this direct TCR–CD1a interaction, in turn preventing T-cell activation. In contrast, some permissive CD1a lipids nestle close to the dimensions of the CD1a antigen cleft, which consequently allows TCR–CD1a engagement and T-cell activation with proinflammatory cytokine secretion and T-cell proliferation. (Created with BioRender.com)

In contrast to TCR–MHC–peptide interactions, some forms of CD1a-mediated T-cell activation are considered to involve an ‘absence of interference’.^34^ In this model, ‘permissive’ activating CD1a ligands are those that can be almost fully sequestered within the CD1a antigen-binding pocket (Figure 1). This permits the TCR to directly interact with the external CD1a surface and promote autoreactive T-cell activation. In contrast, ‘nonpermissive’ inhibitory CD1a ligands can possess large headgroups, in turn leading to partial protrusion of the lipid above the antigen-binding pocket plane to obstruct direct TCR–CD1a docking. In particular, very-long-chain sphingomyelins (SM24:0 and SM24:1), which are endogenous nonpermissive ligands, have been found to be preferentially captured by CD1a and act as natural inhibitors of CD1a-reactive T-cell activation.^28^ It may be that other models of lipid-specific recognition will also emerge.

Between 5% and 10% of cutaneous T cells are CD1a-reactive.^35^ In addition, CD1a-reactive T cells are found within peripheral blood and can be recruited into the skin through cutaneous homing receptors such as CLA, CCR4, CCR6 and CCR10.^36^ Interestingly, unlike conventional MHC-restricted T cells, CD1a-reactive T-cell responses do not appear to always be limited to specific CD1a-lipid combinations resulting in the potential for different lipid antigens to serve as universal CD1a ligands.^34^ Additionally, through the presentation of mycobacterial-derived antigens like dideoxymycobactin and lysyl-phosphatidylglycerol (lysyl-PG) expressed by gram-­positive bacteria, CD1a-reactive T cells likely contribute to host pathogen defences.^13,26,37,38^ The contribution of CD1a in mycobacterium tuberculosis recognition was shown using a group 1 CD1 transgenic mouse model.^39^ Moreover, CD1a-reactive T cells can produce a diverse panel of cytokines and can contribute to cutaneous inflammation under certain circumstances.^10,14,35,37,40–44^ CD1a-reactive T cells are thought to largely express an αβ TCR, but subsets can express a γδ TCR.^45^ In addition, they may express either a CD4 or CD8 co-receptor. With growing evidence to suggest a broad and critical role for CD1a-dependent mechanisms in inflammatory skin disease pathology, we review here the current literature surrounding the role of CD1a in psoriasis, atopic dermatitis (AD) and allergic contact dermatitis (ACD) pathology.^10,40–43^

## Psoriasis

Psoriasis is a common, chronic inflammatory skin disease with an estimated prevalence of between 1% and 3% worldwide.^46^ Multiple forms of psoriasis are recognized, with the most common subtype being psoriasis vulgaris, which accounts for 90% of all people with the condition, in addition to guttate psoriasis and palmoplantar psoriasis.^47^ Classical psoriasis vulgaris lesions are well demarcated, scaly and erythematous, often with a symmetrical extensor distribution. Psoriasis has many systemic associations, including metabolic syndrome, nonalcoholic fatty liver disease, arthritis and bowel disease.^48^ The pathogenesis is multifactorial with a strong genetic component. Over 100 genes conferring increased psoriasis susceptibility have been identified and include those involved in the immune response and barrier integrity.^49–51^ Environmental factors, including infections, medications, such as interferon (IFN)α, and trauma (the Koebner phenomenon), can trigger psoriatic flares.^52–55^

The immunological basis for psoriasis development involves both innate and adaptive immune arms.^54,56–60^ Notably, the adaptive response is primarily T-cell driven with major involvement of activated type 1, type 17 and type 22 responses, plus dysfunction of regulatory T cells.^54,55^ Of the interleukin (IL)-17 subtypes, IL-17A and IL-17F have most notably been shown to be increased in both the blood and lesional skin compartments of patients with psoriasis.^55,61,62^

**Figure 2:**
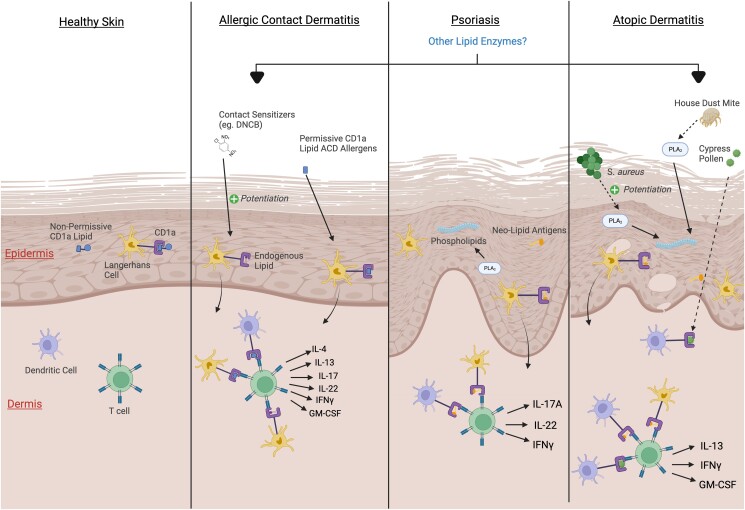
Postulated mechanisms of CD1a involvement in inflammatory skin disease pathogenesis. In healthy skin, the CD1a binding cleft contains a range of nonpermissive and permissive CD1a lipids that balance immune homeostasis. In allergic contact dermatitis (ACD), permissive ACD lipids can directly load onto CD1a expressed by epidermal Langerhans cells, dermal dendritic cells or other cells. Additionally, contact sensitizers such as 2,4-dinitrochlorobenzene (DNCB) may potentiate the production of permissive CD1a lipids in ACD. Together, the presentation of these permissive CD1a lipids contributes to the activation of CD1a-reactive T cells leading to broad cytokine secretion. In psoriasis and atopic dermatitis (AD), phospholipids can be degraded by endogenous or exogenous phospholipase A2s (PLA_2_G4D and acyloxyacyl hydrolase in psoriasis, house dust mite PLA_2_ in AD) and other enzymes to generate permissive CD1a neolipid antigens that promote CD1a-reactive T-cell activation. Additionally, *Staphylococcus aureus* can potentiate host PLA_2_ activity, likely resulting in the production of many additional permissive CD1a lipids. Furthermore, lipids from cypress pollen could be presented by CD1a^+^ dendritic cells to further exacerbate inflammation. GM-CSF, granulocyte-macrophage colony-stimulating factor; IFN, interferon; IL, interleukin (Created with BioRender.com).

The role of phospholipase A_2_ (PLA_2_) in CD1a-dependent pathways was first identified using bee and wasp venom.^63^ PLA_2_s are enzymes that digest phospholipids at the sn-2 position to produce free fatty acids and lysophospholipids, and these were shown to induce CD1a-reactive T-cell activation.^63,64^ The relevance of PLA_2_ in psoriasis was established in subsequent studies investigating PLA_2_G4D, a cytosolic PLA_2_ that is upregulated in psoriasis lesions.^65^ It was shown that the endogenous cytosolic PLA_2_ (PLA_2_G4D) could similarly stimulate CD1a neolipid antigen generation and augment psoriatic inflammation.^40^ PLA_2_G4D-responsive CD1a-reactive T cells secreted IFNγ, IL-17A and IL-22 (Figure 2). Additionally, PLA_2_G4D was found to be transferred through exosomes to surrounding CD1a^+^ cells in response to IFNα stimulation. Moreover, increased frequencies of exosome-responsive CD1a-reactive T cells were detected in psoriasis lesions.^40^

Furthermore, acyloxyacyl hydrolase (AOAH), which primarily acts to metabolize lipopolysaccharides,^66,67^ was later identified to possess PLA_2_ activity.^68^ AOAH expression was found to be upregulated in lesional psoriatic skin relative to nonlesional and healthy skin.^44^ AOAH was discovered to induce the production of CD1a neolipid antigens in a PLA_2_-dependent manner resulting in CD1a-reactive T-cell activation with enhanced IFNγ and IL-22 secretion.^44^ As other PLA_2_s are also upregulated in psoriasis, this ligand generation mechanism may make a broad and important contribution to disease pathology.^69,70^

It has long been known that infection by Group A streptococcal (GAS) species such as *Streptococcus pyogenes* can precede the development of guttate psoriasis, although other psoriatic subtypes are also associated.^71^ Despite this, the mechanistic relationship remains poorly understood. Recently, it was shown that activated GAS-responsive CD1a-reactive T cells were enriched in patients with psoriasis and were predominantly of the type 17 subtype, with a proportion of these being responsive to lysophosphatidylcholine, which is a known permissive endogenous CD1a ligand.^35^ Experiments performed using TCR-transgenic T cells generated via homology-directed repair confirmed these GAS-specific CD1a-reactive T-cell responses to be TCR dependent. As mice do not express CD1a, Chen *et al.* utilized a humanized CD1a-transgenic (hCD1a-Tg) mouse model to demonstrate the characteristic histological features of psoriasis (hyperplasia of the epidermis and dermis with pronounced rete ridges) induced by the presence of GAS.^35^

A commonly used mouse model for psoriasiform inflammation involves the topical application of imiquimod. Imiquimod is used in the management of both precancerous and cancerous skin lesions, but through its agonistic effects on Toll-like receptor (TLR)7 and TLR8 signalling can induce psoriasis-like reactions that can be recapitulated as a type 17 inflammatory phenotype in mice.^72^ By employing this imiquimod approach, hCD1a-Tg mice demonstrated epidermal hyperplasia, exaggerated oedema, erythema and scaling that were suppressed through anti-CD1a blockade.^10,17^ Here, CD1a inhibition was associated with a reduction in granulocytes and production of IL-17A, IL-17F and IL-22 by T cells.

Furthermore, Kim *et al.* compared their findings with human psoriatic samples and discovered upregulated IL-17 and IL-22 production relative to controls, with anti-CD1a antibody administration suppressing this response.^10^ A separate study by Hardman *et al.* employing the imiquimod model in hCD1a-Tg mice revealed that the presence of CD1a could also promote systemic inflammatory responses and that these could similarly be suppressed through anti-CD1a-blocking antibodies.^17^ The cellular alterations recorded included increases in the Langerhans cell and neutrophil levels in the hCD1a-Tg variants compared with wildtype (WT) mice, with this immune cell enrichment suppressed through CD1a inhibition.

## Atopic dermatitis

AD is a common, chronically relapsing, pruritic skin disease.^73^ AD is estimated to affect 20% of children and 10% of adults in the UK.^74^ AD has strong associations with other atopic disorders, such as allergic rhinitis, food allergies and some forms of allergic asthma.^74^ In the past few decades, there has been a two- to threefold increase in AD incidence.^75^ The clinical AD phenotype is usually characterized by pruritic, dry, erythematous lesions with possible excoriations, lichenification and fissuring. Acute lesions tend to possess a more dominant papular–vesicular morphology, whereas chronic lesions can feature nodules and plaques.^76^ Nevertheless, the clinical presentation can be highly heterogeneous.

Immunologically, T cells contribute to both acute and chronic AD inflammation. The acute phase is dominated by type 2 cells that secrete IL-4, IL-5, IL-13 and IL-31.^77,78^ In particular, IL-4 and IL-13 contribute to increased IgE class switching and endothelial adhesion molecule expression, with negative regulation of epidermal differentiation proteins and antimicrobial peptides.^79–81^ In chronic atopic cutaneous inflammation, a more mixed type 1/2/17/22 cytokine milieu is present, for example through the secretion of mediators such as IFNγ that upregulates MHC II and intercellular adhesion molecule-1 expression, as well as type 1 and type 2 chemokines.^82–86^ Additional contributions from type 17, type 22 and type 9 T cells have also been described.^79,87–92^

A broad range of factors contribute to the pathogenesis of AD, including genetics, environmental agents, immune dysregulation and infections. Importantly, loss-of-function mutations in *FLG*, which encodes an integral epidermal barrier protein, strongly predisposes to AD development.^93^ Filaggrin has a number of cutaneous roles including the maintenance of cutaneous hydration levels, barrier integrity, skin acidity and antimicrobial defences.^94–98^

The importance of CD1a in AD was first established through the discovery of peripheral blood and cutaneous house dust mite (HDM)-specific CD1a-reactive T cells, which were enriched in patients with AD compared with controls.^41^ Interestingly, the presence of null *FLG* mutations was correlated with an increase in HDM-specific CD1a-reactive T cells. In addition, HDM-responsive CD1a-reactive T cells were associated with disease severity, total IgE titres and secretion of IL-13, IFNγ and granulocyte-macrophage colony-stimulating factor (GM-CSF), which are mediators that are implicated in AD pathology. Fractionation of the HDM extract revealed that the CD1a-dependent T-cell activation was dependent on a PLA_2_ involved in CD1a neolipid antigen generation, analogous to the mechanisms established for bee venom, wasp venom and endogenous PLA_2_s. Moreover, PLA_2_s have previously been linked with atopic disorders.^99–105^ Exposure to HDM stimulated the cutaneous recruitment of CD1a-responsive T cells and production of various cytokines: IL-4, IL-5, IL-13 and GM-CSF.^41^ Additionally, skin HDM-specific CD1a-reactive T cells responded to bee venom PLA_2_ indicating the presence of multi-PLA_2_-responsive CD1a-reactive T cells in AD.

Another trigger of AD relapse is exposure to environmental pollen. In particular, cypress pollen (containing phosphatidylcholine 18:2/18:2) could be presented by CD1a^+^ dendritic cells, and in turn potentiate T-cell activation in patients who are allergic.^8^

In addition to the imiquimod model of psoriasiform inflammation, a mouse model using MC903 (a low-calcaemic vitamin D3 analogue) is regularly used for studying type 2 pathology.^106^ MC903, which is more commonly known as calcipotriol, is used in the management of psoriasis. It was later observed to sometimes induce dermatitis in a subset of patients. The topical application of MC903 in hCD1a-Tg mice stimulated cutaneous thickening, inflammation and broad immune cell infiltration compared with WT mice, with this response being suppressed by anti-CD1a antibody administration.^17^ Additionally, type 2 and proinflammatory cytokine production were upregulated in hCD1a-Tg mice, which could be inhibited by anti-CD1a antibodies.

Additionally, *Staphylococcus aureus* has long been associated with AD. One study revealed that 70% of lesional AD skin and 39% of nonlesional skin were colonized by *S. aureus*.^107^ Despite this, the relationship between *S*. *aureus* and AD remains poorly understood. The discovery of increased CD4^+^ T cells that react to CD1a–lysyl-PG, a characteristic *S. aureus* lipid, in patients with AD may potentially help to explain this association.^37^ Expression profiling of lysyl-PG responsive T cells revealed marked upregulation of IL-4, IL-5 and IL-13 alongside other AD-relevant mediators such as GM-CSF and tumour necrosis factor.^37^ Moreover, acute AD lesions contain elevated ILC2 numbers that secrete type 2 mediators.^108^ Thymic stromal lymphopoietin is a pleiotropic cytokine that is enriched in AD lesions and was shown to induce ILC2 expression of CD1a, suggesting the presence of a possible positive feedback loop that may exacerbate atopic cutaneous inflammation.^14,109^ Therefore, there is a growing evidence base to support the broad role of CD1a within AD pathogenesis.

## Allergic contact dermatitis

The development of ACD is dependent on cutaneous exposure to a sensitizing allergen. ACD allergens are commonly found in metals, fragrances, cosmetics, dyes and preservatives. Clinically, ACD lesions are characterized by erythema, blistering, oedema and pruritus at the site of allergen exposure.^110^ Historically, AD was thought to protect against the development of ACD;^110^ however, subsequent evidence has suggested the reverse, potentially because of a combination of increased skin permeability, dysregulation in shared immune response pathways and the application of topical medications that may contain ACD allergens.^110,111^

ACD is considered a type IV hypersensitivity response under the Gell and Coomb classification through association with a delayed T-cell response. Mechanistically, the conventional model of ACD is thought to involve two separate phases: sensitization and elicitation.^112^ Haptenation of ACD allergens through complexing with cutaneous carrier proteins has been thought to be required for immune recognition, prior to internalization by APCs and presentation via MHC molecules to induce the development of memory and effector T cells.^112,113^ Subsequent allergenic exposure triggers rapid and heightened inflammation through stimulation of these presensitized T cells. Nevertheless, many known ACD allergens are unlikely to undergo haptenation, suggesting that alternative mechanisms may contribute to disease pathology.^114^ Type 1 cells are the primary drivers of ACD pathogenesis although type 2, type 17 and type 22 cells also make important contributions.^110,115–118^

Recent evidence indicates that CD1a-dependent mechanisms are involved in ACD development. First, urushiol, which is found within poison ivy and historically considered to be a hapten, was shown to directly induce CD1a-dependent T-cell responses.^10,119^ Using a hCD1a-Tg mouse model, Kim *et al.* discovered that skin inflammation was driven by urushiol-specific CD1a-dependent CD4^+^ T cells with secretion of IL-17 and IL-22.^10^ This characteristic type 17 cell signature corresponded to the cytokine changes observed in humans with poison ivy dermatitis. Urushiol demonstrated the capacity to displace ganglioside GD_3_ from the CD1a cleft, with potentially 20% of the urushiol molecule extending outside of the cleft.

The known repertoire of CD1a contact dermatitis allergens was extended through the discovery of farnesol, benzyl benzoate, benzyl cinnamate and coenzyme Q-compounds.^43^ These allergens were found to not require cellular processing prior to CD1a loading. Additionally, Nicolai *et al.* determined that farnesol could reside deeply within the CD1a cleft, adopting a medial alignment and suggesting that haptenation is not essential.^43^ Similar to urushiol, farnesol was observed to displace endogenous lipids from the CD1a groove, further supporting a hapten-independent model of ACD pathogenesis. Moreover, multiple additional contact sensitizers were shown to potentiate CD1-specific responses that were dependent on the presence of endogenous lipids, with 2,4-dinitrochlorobenzene (DNCB) in particular, inducing activation of CD1a-reactive T cells.^120^ This resulted in enhanced secretion of IFNγ, GM-CSF, type 2 and type 22 mediators. These cells were also shown to express CLA and CCR4, suggesting that they may possess the capacity to infiltrate into the skin. Therefore, allergen-­sensitive CD1a-reactive T cells contribute to ACD development. Together, this expands upon the long-recognized model of ACD involving haptenation and a type IV hypersensitivity response.

## Therapeutic potential and conclusions

Many different treatments are now available for inflammatory skin diseases that have emerged through an improved understanding of disease pathogenesis. Therapies can effectively and specifically target pathological cytokine pathways, although they are not necessarily effective in all patients and specific adverse effects can occur.^121^ Approaches so far have not specifically targeted the upstream elements associated with T-cell engagement with antigen, largely because of uncertainties about the dominant antigenic targets involved. The increased understanding of the broad and vital role that CD1a plays in the pathogenesis of inflammatory dermatoses reveals a possible novel target. The highly skin-specific expression of CD1a and its contribution to disease pathogenesis suggests that targeting CD1a-dependent pathways may be an effective approach to treat inflammatory cutaneous disorders while minimizing side-effects. Preclinical studies are encouraging^10,17^ and an antibody is completing Phase I development with an early focus on AD (https://classic.clinicaltrials.gov/ct2/show/NCT04668066). However, the antibody binds a region of CD1a that is flexible and is known to be influenced by the nature of the housed lipid.^25^ It is not known whether this might have an impact on efficacy, but regardless of whether this initial trial is successful, the preclinical and clinical studies will uncover further biology that will help define risk–benefit and optimal future therapeutic strategies.

## Data Availability

No new data generated.

## CPD questions

### Learning objective

To understand how CD1a contributes to inflammatory skin disease development.

### Question 1

Which epidermal cell type expresses CD1a at constitutively high levels?

(a) B cells.
(b) Keratinocytes.
(c) Langerhans cells.
(d) Melanocytes.
(e) T cells.

### Question 2

Which class of nonpermissive lipids have been shown to be selectively captured by CD1a?

(a) Cholesterol.
(b) Short-chain sphingomyelins.
(c) Squalene.
(d) Triacylglycerides.
(e) Very-long-chain sphingomyelins.

### Question 3

What type of phospholipase A_2_ (PLA_2_) was shown to ­contribute to psoriasis development in a CD1a-dependent manner?

(a) Bee venom PLA_2_.
(b) House dust mite PLA_2_.
(c) Lipoprotein-associated PLA_2_.
(d) PLA_2_G4D.
(e) Wasp venom PLA_2_.

### Question 4

In human transgenic mouse models, which of the following substances can be used to recapitulate type 17- and type 2-cytokine inflammation, respectively?

(a) House dust mite and MC903.
(b) Imiquimod and MC903.
(c) MC903 and imiquimod.
(d) Urushiol and imiquimod.
(e) Urushiol and MC903.

### Question 5

Which CD1 lipid antigen has been associated with poison ivy dermatitis?

(a) Cholesterol.
(b) Farnesol.
(c) Sphingomyelin.
(d) Squalene.
(a) Urushiol.

## Instructions for answering questions

This learning activity is freely available online at https://oupce.rievent.com/a/JTUDZI

Users are encouraged to

- Read the article in print or online, paying particular attention to the learning points and any author conflict of interest disclosures.
- Reflect on the article.
- Register or login online at https://oupce.rievent.com/a/JTUDZI and answer the CPD questions.
- Complete the required evaluation component of the activity.

Once the test is passed, you will receive a certificate and the learning activity can be added to your RCP CPD diary as a self-certified entry.

This activity will be available for CPD credit for 5 years following its publication date. At that time, it will be reviewed and potentially updated and extended for an additional period.
